# Assessment of the Level of Pain Intensity and the Level of Anxiety Treated as State and Trait in Patients with Osteoarthritis of the Limbs

**DOI:** 10.1155/2020/5904743

**Published:** 2020-04-23

**Authors:** Jadwiga Kuciel-Lewandowska, Michał Kasperczak, Łukasz B. Lewandowski, Małgorzata Paprocka-Borowicz

**Affiliations:** Department of Physiotherapy, Medical University of Wroclaw, Wrocław, Poland

## Abstract

**Introduction:**

Osteoarthritis of the musculoskeletal system is accompanied with chronic pain which is the main factor in mood lowering, causing anxiety. Rehabilitation conducted in the framework of spa therapy and outpatient care aims at eliminating or reducing pain and improving physical fitness. Pain relief is an expected phenomenon because it improves the quality of life. *Aim of the study*. The aim of the study was to evaluate the effect of rehabilitation in the spa and in outpatient clinic on the level of pain and anxiety in patients with degenerative joints and disc disease. *Material and methods*. The study included a comprehensive treatment conducted in the spa and in outpatient clinic. Observation included 120 persons with disorders of the musculoskeletal system treated in the spa Przerzeczyn-Zdrój. The second group of patients was treated in the rehabilitation clinic. The examinations were performed before and after treatment. The scope of the observations included self-evaluation of anxiety treated as a state and a trait, the level of intensity of pain, medical history, and sociodemographic background interview. In the observations, there were VAS scale and State Trait Anxiety Inventory STAI used.

**Result:**

As a result of the spa therapy and therapy performed in an outpatient clinic, there was an improvement in lowering the level of pain and anxiety noted.

**Conclusions:**

1. Spa therapy and treatment performed in an outpatient clinic reduce the level of pain and anxiety in patients with degenerative disease of the musculoskeletal system. 2. It was found that the therapy conducted in the spa was more effective in lowering the level of pain and anxiety. This trial is registered with NCT03405350.

## 1. Introduction

The basic method of spa treatment is balneotherapy. The essence of spa treatment is a complex medical procedure involving the use of physical therapy, psychotherapy, diet, and medication if necessary. The modern spa treatment differs significantly from that used in the recent past. There were medical equipment of the latest generation and modern methods introduced. An integral part of spa treatment is also psychotherapy and health education. Spa therapy is characterized by complexity and diversity which determine the effectiveness of treatment in many chronic diseases [1]. Spa therapy has great potential for relaxation activities and thus affects the emotional state of patients. Both the improvement of mood as well as reduction or disappearance of anxiety and pain are the most common consequences of treatment in the spa. Contact with nature, relief of symptoms, complete healing, or reduction of the symptoms of the disease may become a source of well-being and feeling of joy of life. This is sometimes of unattainable level in other therapies. Osteoarthritis of the musculoskeletal system accompanied by chronic pain is the main factor in reducing the mood and causing anxiety. Analysis of correlation between pain and anxiety showed a statistically significant relationship [2]. Rehabilitation conducted in the framework of spa treatment and ambulatory care aims at eliminating or reducing pain and improving physical fitness. Pain relief is a phenomenon expected because it improves the quality of life. Rehabilitation in any form is curative bringing distant beneficial effects without burdening the system with side effects of the actions.

The aim of the study was to evaluate the effect of rehabilitation in the spa and in outpatient clinic on the level of pain and anxiety in patients with degenerative joints and disc disease.

## 2. Materials and Methods

The study was conducted in spa Przerzeczyn-Zdroj and Outpatient Rehabilitation Clinic Stabłowice in Wroclaw. The spa observation consisted of patients undergoing complex therapy within the 21-day sanatorium stays. Group A consisted of *n* = 120 spa patients suffering from pains caused by degenerative joints and disc disease. The age of patients ranged between 29 and 78 years, whereas the average age was 54.8 years. Among the subjects, there were 78 women and 42 men. The study inclusion criterion was the presence of chronic pain resulting from degenerative joints and disc disease or discopathy, the patient's consent to participate in the study, the lack of a history of a past anxiety-depression incident requiring medication, and age below 80 years. The exclusion criteria from the study were lack of chronic pain syndrome, previous anxiety-depression incident requiring medication, and lack of consent to participate in the study. Patients received a series of 10 treatments of different types of therapy depending on the needs and reported problems. A typical set of procedures used in the treatment covered radon-sulfide baths, mud wraps, both group and individual therapeutic gymnastics in the gym and in the pool, laser radiation biostimulation, and interference currents. Any changes concerned only sets treatments from the physiotherapy department. The treatments were performed every second day interchangeably. The number of prescribed, during the stay, individual treatments was 10. The patients used the help of a psychologist and nutritionist and were covered by a health education program, including various forms of relaxation activities such as gymnastics and therapeutic field walks, Nordic-walking, music therapy. Outpatients in group B consisted of patients treated from pain resulting from degenerative joints and disc disease or discopathy. In group B, there were 63 people in the age ranging from 37 to 79 years, whereas the mean age was 55.5 years, 18 men and 45 women. In this group, patients received a series of 10 physiotherapeutical treatments, dry massage, and therapeutic gymnastics. The treatments were performed daily. Patients did not have pool gymnastics, therapeutic baths, therapeutic field walks, psychologist sessions, and health education. They carried out their regular private and professional activities. Group B was subject to the same inclusion and exclusion criteria in the study.

The scope of the observations included the assessment of pain intensity and self-evaluation of anxiety as a state and as a trait. In the observations, there were two measurement tools used: VAS scale and a standard Self-State and State Trait Anxiety Inventory STAI-C. D Spielberger, J. Strelau, M. Tesarczyk, and K. Wrzesniewski.

The STAI questionnaire enables detection of people with significantly low or high levels of anxiety understood as a “constant inner disposition” (trait). It also allows the researcher to record changes in the severity of anxiety assessed as a state that occurs under the influence of certain external stimuli. Anxiety-state is characterized by high volatility; it is a subjective phenomenon, susceptible to the influence of external factors especially threatening factors, and it is associated with the activation of the autonomic nervous system. On the other hand, anxiety as a trait is acquired and very stable, and it makes a person susceptible to excessive anxiety reactions in relation to events and situations which are not endangering. The questionnaire includes a short instruction informing about the nature of the study and its execution method.

It consists of two scales: STAI X1 is used to examine the anxiety-state, whereas scale STAI X2 to evaluate anxiety-trait. In each of them, there are 20 statements, and the person being tested should respond to them by ticking the digits from 1 to 4, describing subjective feelings in the most accurate way. To evaluate and calculate the results, there is a key used that does not allow the examiner for a simple summation of numerical values included in the test, and it is necessary to apply another method according to the following principles: 1 = 4, 2 = 3, 3 = 2, 4 = 1. The key contains all the rules of conversion and is available only for the examiner. The first so-called raw results of both scales may be in the range of 20 points (low anxiety) to 80 points (high level of anxiety). The results are mapped to tables with the so-called percentile ranks and sten scores, which in turn are assigned to gender and age. In the study, only raw results were used due to the fact that patients were not divided into groups depending on gender and age. The obtained median value was evaluated in both groups.

In the study, there was a standard VAS scale used. The visual-analog scale of pain assessment (VAS) is a graphic and descriptive scale used in the evaluation of pain intensity, where to the level of pain, there is assigned a numerical scale from 0 to 10, 0 denoting no pain, whereas 10 denoting very strong pain. VAS is the most commonly used measurement tool in the assessment of pain intensity.

The study was performed in both groups before and after treatment. The results were statistically analyzed with the use of STATISTICA 9.1 program in Polish version. The value received ‘before treatment' and ‘after treatment' for one patient was compared. In the statistical significance, the level was set at *p* = or <0.05. Descriptive statistics were conducted. To assess the significance of differences of the obtained results, there were two tests used: the Sign test and Wilcoxon test. The results are presented in tables and in the form of a bar chart.

The study was approved by the Bioethics Committee of the Medical University in Wroclaw-Opinion No KB-401/2008; there was also written consent of the patients and of the directors of the spa and the outpatient clinic obtained.

## 3. Results

As a result of the therapy conducted in the spa and in an outpatient clinic, there was improvement in lowering the level of pain and anxiety. Tables 1 and 2 show the observed changes in the level of anxiety in the two groups, whereas Table 3 depicts changes in the level of pain.

**Figure 1:**
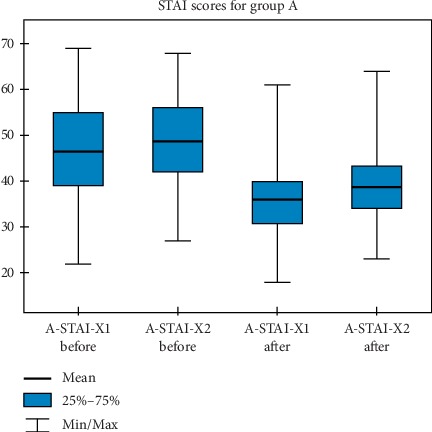
STAI-group A results.

**Figure 2:**
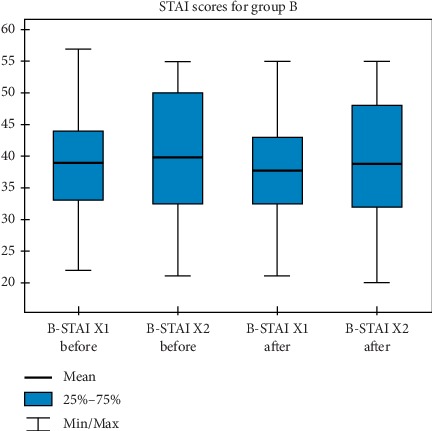
STAI-group B results.

**Figure 3:**
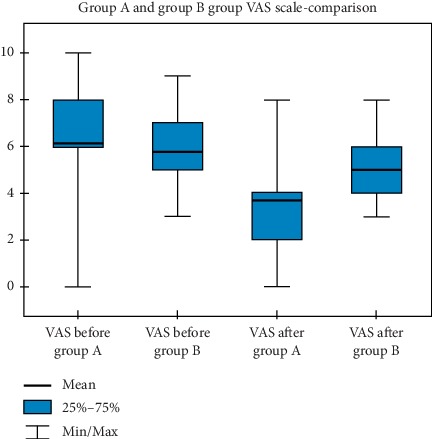
VAS scale-group A and group B group comparison.

Figures 1–3 show graphical charts of the observed changes.

## 4. Discussion

In the conducted study, the changes in the level of anxiety and pain, under the influence of spa therapy and ambulatory care, were observed. The STAI questionnaire used in the study allows the researchers to record changes in the level of anxiety caused by the influence of other variables, whereas the X-1 scale evaluates anxiety globally. The obtained average raw results' value of both scales showed average level of anxiety in the observed groups. After treatment, there was a noticeable decline in these values; thus, the change was in the direction of reducing the level of anxiety, and it was also noted in case of VAS that the level of pain declined. The group treated at the spa obtained a better result. This difference may be the result of a comprehensive treatment provided in the spa including psychotherapy, relaxation, and educational activities.

Pain, especially chronic pain, as physical and psychological stressor, affects the disposition and mood and lowered mood and affects the perception of pain intensity. Thus, the emergence of chronic illness can be considered as a traumatic factor for the patient. Chronic diseases are potent stressors not only because of the excessive length of their duration but also of the intensity of therapy and the accompanying sensations of pain arising from the diagnostic, therapeutic procedures, and the essence of the disease itself.

The ability to recognize and deal with the experiences associated with the disease, especially negative emotions, seems to be a major effort in overcoming the disease [3]. Illness as a stress factor can cause various psychological reactions: anxiety and depression, usually in the form of low mood, and aggression [4]. There is often observed the comorbidity of mood and anxiety disorders of somatic diseases especially in the field of cardiology, dermatology, oncology, pulmonary, metabolic diseases (diabetes), and the musculoskeletal system illnesses [5–7].

Both chronic pain and the accompanying anxiety, lowered mood, or depression are the onerous and debilitating ailment in the population. Coexistence of these symptoms limits functional state and primarily impairs the physical, mental, and social development [8–10]. Difficulties in the healing at the same time increase the cost of the treatment without the clear progress in therapy. The possibility of the occurrence of pain and lowered mood, and in the consequence anxiety, may result from neurochemical transduction disorders. Factors such as female gender, cancer, pain, the presence of the disease limiting normal function, smoking, depression, mental disorders, and pessimism determine the intensity and duration of anxiety. Also, among the factors which are the subject of anxiety during the therapy there is fear of the unknown mentioned. The presence of the disease itself is a serious burden on the physical and mental health because of the discomfort and the accompanying negative emotions, which greatly limit daily functioning [11].

Research carried out by Lelonek and Cieslik confirmed the dependence of the level of anxiety and disease duration. This factor may predispose to increased anxiety-state and anxiety-state, and the longer the duration of the disease, the higher the level of anxiety as a state and a trait [12]. It is important, thus, to shorten the duration of illness. In the case of diseases of the musculoskeletal system, it is particularly important to fight with pain and disability. From 30% to 80% of patients with chronic pain suffer from depression and anxiety. Such study was conducted in both psychiatric clinics and at family doctor practice. The largest group of patients with chronic pain complicated by anxiety and depression were, however, patients of orthopedic, rheumatological, and dental clinics, and higher levels of pain intensity correlated with a higher incidence of anxiety and lowered mood or even depression. The main cause of pain and related emotional dysfunctions were obviously musculoskeletal diseases [13–15].

At the same time, it is crucial to emphasize the diagnostic difficulties of atypical depression and appearance of “masking” of the depression process in the form of chronic feeling of pain which complicates diagnosis and therapy [16–18].

Research by Talarowski-Bogusz et al. conducted in patients with osteoarthritis of the spine and hips have shown that the use of physical treatments such as low magnetic field and biostimulating laser causes a reduction of pain in patients with anxiety, depression, and lowered mood. Moreover, in these patients, the reduction of the intensity of pain sensations alleviates the intensity of the symptoms of depression and anxiety only in case of mild-to-moderate severity [19].

Given the mutual dependence of pain, anxiety, and lowered mood and involvement of the particular structures of the brain and neurotransmitters in the appearance of these conditions, it appears necessary to implement a complex therapy both in terms of medication, psychotherapy, and other techniques. It also seems that a spa therapy has a lot to offer, being a response to dehumanized modern medicine. Currently, there is a huge need for a holistic recognition of a man, his state of health, and disease. Conditions in the spa can be a curative factor in itself in terms of infrastructure and the selection of competent and sympathetic staff. Spa treatments meet the criteria for a holistic model of therapy. Regardless of the methods recommended in the spa, patients treated there have time to rest and cultivate relationships with people, nature, beautiful landscape, and pull away from the home or work environment to change health habits especially in terms of diet, physical activity, and the fight against addictions and pain [20–23].

## 5. Conclusions

1. Spa therapy and treatment performed in an outpatient clinic reduce the level of pain and anxiety in patients with degenerative disease of the musculoskeletal system
2. Greater efficacy in reducing pain and anxiety was found in patients treated at the spa

## Figures and Tables

**Table 1:** The values obtained for descriptive statistics in the assessment of STAI questionnaire-scores for group A.

	N	Mean	Standard deviation	Minimum	Maximum	Percentiles		
						25.	50.	75.
A-STAI X1 before	120	46,42	10,42	22,00	69,00	39,00	45,00	55,00
A-STAI X2 before	120	48,80	9,55	27,00	68,00	42,00	49,00	56,00
A-STAI X1 after	120	35,93	8,37	18,00	61,00	30,75	35,00	40,00
A-STAI X2 after	120	39,72	7,86	23,00	64,00	34,00	40,00	43,20

N, number of trials. A, group. A-STAI X1, anxiety-state. STAI X2, anxiety-trait.

**Table 2:** The values obtained for descriptive statistics in the assessment of the STAI questionnaire-scores for group B.

	N	Mean	Standard deviation	Minimum	Maximum	Percentiles		
						25.	50.	75.
B-STAI X1 before	63	38,90	8,72	22,00	57,00	33,00	39,00	44,00
B-STAI X2 before	63	39,81	9,81	21,00	55,00	32,50	41,00	50,00
B-STAI X1 after	63	37,76	8,46	21,00	55,00	32,50	37,00	43,00
B-STAI X2 after	63	38,76	9,69	20,00	55,00	32,00	39,00	48,00

N, number of trials. B, group B. STAI X1, anxiety-state. STAI X2, anxiety-trait.

**Table 3:** VAS scale-group A and group B.

	N	Mean	Standard deviation	Minimum	Maximum	Percentiles		
						25.	50.	75.
VAS before group *A*	120	6,13	1,77	0,00	10,00	6,00	6,00	8,00
VAS before group *B*	63	5,76	1,67	3,00	9,00	5,00	6,00	7,00
VAS after group *A*	120	3,67	1,46	0,00	8,00	2,00	4,00	4,00
VAS after group *B*	63	5,00	1,26	3,00	8,00	4,00	5,00	6,00

N, number of trials. A, group A. B, group B. VAS, scale.

## Data Availability

All data are contained and described within the manuscript. The datasets used and/or analyzed during the current study are available from the corresponding author on reasonable request.
